# Cycloastragenol Improves Fatty Acid Metabolism Through NHR-49/FAT-7 Suppression and Potent AAK-2 Activation in *Caenorhabditis elegans* Obesity Model

**DOI:** 10.3390/ijms27020772

**Published:** 2026-01-13

**Authors:** Liliya V. Mihaylova, Martina S. Savova, Monika N. Todorova, Valeria Tonova, Biser K. Binev, Milen I. Georgiev

**Affiliations:** 1 Laboratory of Metabolomics, Department of Biotechnology, Institute of Microbiology, Bulgarian Academy of Sciences, 139 Ruski Blvd., 4000 Plovdiv, Bulgaria; m.sav@abv.bg (M.S.S.); mntodorova@yahoo.com (M.N.T.); biser.binev@abv.bg (B.K.B.); milengeorgiev@gbg.bg (M.I.G.); 2 Department of Plant Cell Biotechnology, Center of Plant Systems Biology and Biotechnology, 4000 Plovdiv, Bulgaria; 3 Department of Molecular Stress Physiology, Center of Plant Systems Biology and Biotechnology, 4000 Plovdiv, Bulgaria; tonova@cpsbb.eu

**Keywords:** obesity, lipid metabolism, *Caenorhabditis elegans*, triterpenoids, cycloastragenol, orlistat

## Abstract

Obesity is among the top contributing factors for non-communicable chronic disease development and has attained menacing global proportions, affecting approximately one of eight adults. Phytochemicals that support energy metabolism and prevent obesity development have been the subject of intense research endeavors over the past several decades. Cycloastragenol is a natural triterpenoid compound and aglycon of astragaloside IV, known for activating telomerase and mitigating cellular aging. Here, we aim to characterize the effect of cycloastragenol on lipid metabolism in a glucose-induced obesity model in *Caenorhabditis elegans*. We assessed the changes in the body length, width, and area in *C. elegans* maintained under elevated glucose through automated WormLab system. Lipid accumulation in the presence of either cycloastragenol (100 μM) or orlistat (12 μM), used as a positive anti-obesity control drug, was quantified through Nile Red fluorescent staining. Furthermore, we evaluated the changes in key energy metabolism molecular players in GFP-reporter transgenic strains. Our results revealed that cycloastragenol treatment decreased mean body area and reduced lipid accumulation in the *C. elegans* glucose-induced model. The mechanistic data indicated that cycloastragenol suppresses the nuclear hormone receptor family member NHR-49 and the delta(9)-fatty-acid desaturase 7 (FAT-7) enzyme, and activates the 5′-AMP-activated protein kinase catalytic subunit alpha-2 (AAK-2) and the protein skinhead 1 (SKN-1) signaling. Collectively, our findings highlight that cycloastragenol reprograms lipid metabolism by down-regulating the insulin-like receptor (*daf-2*)/phosphatidylinositol 3-kinase (*age-1*)/NHR-49 signaling while simultaneously enhancing the activity of the AAK-2/NAD-dependent protein deacetylase (SIR-2.1) pathway. The anti-obesogenic potential of cycloastragenol rationalizes further validation in the context of metabolic diseases and obesity management.

## 1. Introduction

The escalation in the global prevalence of being overweight and of obesity have attained epidemic proportions, affecting over 2 billion people worldwide. Projections estimate that obesity rates will double by the end of the next decade, which would mean that almost half of the global population would be threatened by the burden of obesity [1,2]. Correspondingly, obesity-related chronic disease incidences increases, including that of cardiovascular diseases, hypertension, type 2 diabetes, metabolic syndrome, and insulin resistance [3,4,5]. The advancement of multi-receptor drugs that mimic the glucagon-like peptide-1 (GLP-1), glucose-dependent insulinotropic polypeptide (GIP) and glucagon action, including semaglutide, tirzepatide, and retatrutide, has driven the pharmacotherapy of obesity to a new era of effectiveness [6,7]. Nevertheless, the rapid evolvement of this novel drug class has pushed forward the mechanistic obesity research [4,8] and exposed novel anti-obesity targets such as the modulation of key effectors of the phosphoinositide-3-kinase (PI3K)/protein kinase B (AKT)/phosphodiesterase (PDE) signaling pathway [9,10,11]. For instance, pharmacological and genetic inhibition of AXL enhance the thermogenic capacity of brown and white adipocytes through inhibition of the PI3K/AKT/PDE signaling pathway, resulting in the induction of nuclear localization of Forkhead box protein O1 (FOXO1) and increased intracellular cAMP levels via PDE3/4 inhibition [11].

Maintaining metabolic health requires a deeper understanding of the energy homeostasis and the response to high-calorie diets. The contribution of inflammatory–immune reactions in obesity and its related comorbidities have unequivocal rooting in the elevated circulating levels of free fatty acids, reactive oxygen, and nitrogen species. The limited ability of the homeostatic system to manage these challenges leads to insulin signaling dysregulations in adipose, muscular, and hepatic tissues [5,12]. The insulin/insulin-like growth factor (IGF) signaling (IIS) pathway is one of the highly evolutionarily conserved obesity-related pathways. It plays a major part in nutrient metabolism, growth, and development, and in the regulation of body composition throughout the whole lifespan [4,13,14]. Notably, many of the critical metabolic pathways in *Caenorhabditis elegans* are conserved with those in mammals, including the IIS, which aids in understanding the intricate mechanisms of obesity and metabolic disorders in this model organism [13,14,15,16,17,18]. The IIS pathway in *C. elegans* represents a complex gene regulatory network that primarily controls crucial physiological processes, including metabolism, stress resistance, and longevity. The genes and their products interact to interpret environmental signals, like food availability, and modulate gene expression to promote survival [13,19]. The insulin-like receptor (DAF-2) activation triggers a phosphorylation cascade that ultimately represses the Forkhead box protein O (DAF-16) transcription factor and promotes normal growth and development. Another key player of the IIS is the phosphatidylinositol 3-kinase (AGE-1), which acts downstream of the DAF-2. The DAF-2/AGE-1 and the nuclear hormone receptor family member nhr-49 (NHR-49)/mediator of RNA polymerase II transcription subunit 15 (MDT-15) pathways converge to regulate metabolic and stress-response genes, highlighting the interplay between aging and metabolism [19]. Additionally, *C. elegans* has been effectively used to investigate dyslipidemia, endocrine regulation, mitochondria dysfunction, and the effects of pharmacological or dietary interventions on metabolic health [13,20,21,22,23]. The conserved pathways, such as those regulated by the NAD-dependent protein deacetylase (SIR-2.1), the 5′-AMP-activated protein kinase catalytic subunit alpha-2 (AAK-2), the target of rapamycin homolog (LET-363) and the orthologue of the mammalian nuclear factor erythroid 2-related factor 2 (NRF2) the protein skinhead 1 (SKN-1), play crucial roles in cellular metabolism and mitochondrial function, linking nutrient sensing to energy homeostasis and longevity [13,14,18,24,25,26,27].

Phytochemicals that support energy metabolism and prevent obesity development have been the subject of intense research over the past several decades [20,28,29,30,31]. Obesity and lipid dysregulation are closely linked to impaired mitochondrial function and oxidative stress, and natural compounds isolated from *Astragalus* species have been shown to modulate these pathways [30,31]. Cycloastragenol is a natural triterpenoid compound and the aglycon of astragaloside IV, known for activating telomerase and mitigating cellular aging [25,32,33,34]. The telomerase activity contributes to the extension of somatic cell lifespan and is mediated through the regulation of the mitogen-activated protein kinase (MAPK) and protein kinase B (AKT) signaling pathways, which are key regulators of cellular proliferation, differentiation, motility, survival, and stress responses. In line with its telomerase-activating properties, several studies have highlighted the broader role of cycloastragenol in mitigating age-related cellular decline, counteracting fibrosis and activating autophagy [25,30,35,36,37]. Consistent with this, cycloastragenol was shown to modulate longevity-related pathways such as the fibroblast growth factor 1 receptor (FGFR1)/telomerase reverse transcriptase (TERT)/Klotho axis, enhancing β-Klotho expression, reducing oxidative stress, and preventing apoptosis in primary granulosa cells [38]. Supplementation with cycloastragenol alleviates hepatic lipid accumulation, lowers serum triglycerides and glucose levels through activation of the farnesoid X receptor (FXR) signaling pathway, indicating that it is capable of lipid metabolism modulation in mice [39]. Cycloastragenol treatment in a diabetic kidney disease murine model mitigated mitochondrial oxidative overload and renal tubular injury through stimulation in the transcriptional factor EB (TFEB) nuclear translocation [40]. These findings highlight the potential of the triterpenoid compound to modulate metabolic diseases. However, research on the effect of cycloastragenol on lipid metabolism and obesity remains limited.

Collectively, these data suggest that cycloastragenol acts as a multi-target natural compound capable of interacting with various receptors to regulate energy homeostasis and lipid metabolism, reinforcing the need for further in-depth investigation to clarify its mechanism of action in obesity and metabolic diseases models. Therefore, in the present study we evaluated the anti-obesity potential of cycloastragenol in a glucose-induced model in *C. elegans* and explored the molecular networks involved in its lipid-reducing activity.

## 2. Results

### 2.1. Cycloastragenol Diminished Glucose-Induced Lipid Accumulation in C. elegans

**Figure 1:**
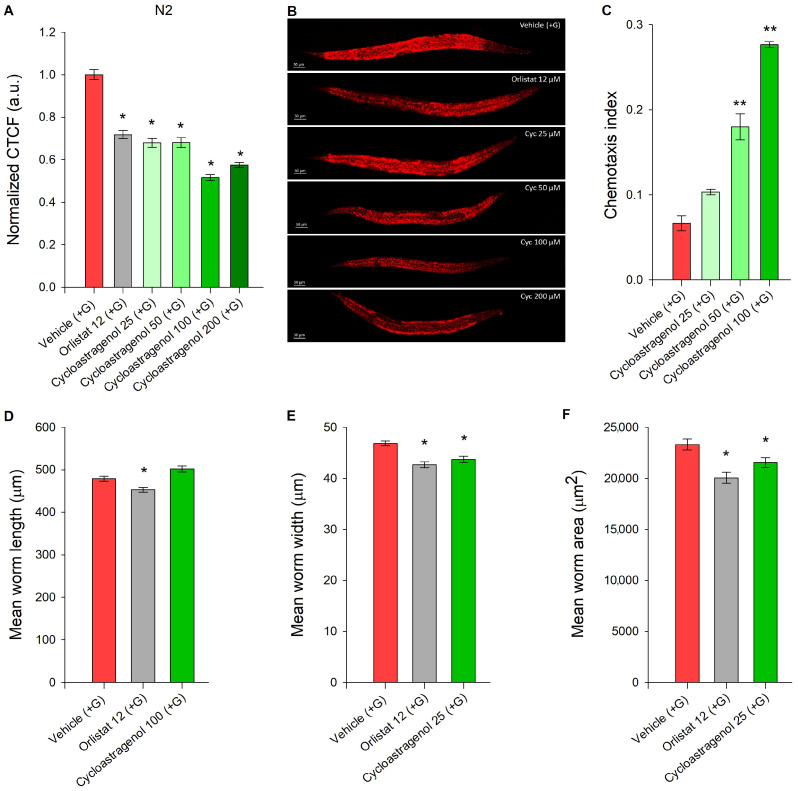
Cycloastragenol decreases lipid accumulation, acts as a chemoattractant, and modulates body parameters in glucose (+G)-supplemented nematodes. (**A**) Quantification of triglyceride accumulation (Nile red lipid staining) in wild-type N2 strain nematodes upon cycloastragenol (25–100 μM) supplementation in obesity (+G) model. Data are represented as corrected total cell fluorescence (CTCF) normalized to the vehicle (+G) group in arbitrary units (a.u.); mean ± SEM, n = 90. (**B**) Representative images of Nile red-stained nematodes, 20× magnification, scale bar (50 μm). (**C**) Chemotaxis index of nematodes, treated with cycloastragenol (25–100 μM), n = 300–600, represented as mean ± SEM; significance level set at ** *p* < 0.01, via one way ANOVA, followed by Tukey’s post hoc test. (**D**) Mean length, (**E**) width, and (**F**) area were measured and analyzed using the WormLab software (version 2023.1.1). Data presented as mean ± SEM (n = 110–130). For (**A**,**D**–**F**) statistical differences were evaluated by ANOVA on ranks followed by Dunn’s post hoc test * *p* < 0.05.

Elevated glucose content in the nematode growth media results in the increase in lipid droplet accumulation in *C. elegans* [16]. Orlistat is an anti-obesity medicine commonly used as a reference compound for its lipid-reducing effect [20]. In our earlier studies, we have demonstrated the potential of triterpenoid compounds (betulinic acid and maslinic acid) to modulate nutrient-sensing signaling networks such as the phosphoinositide 3 kinase (PI3K)/protein kinase B (AKT) signaling in both in vitro and in vivo models of obesity [10,20,41]. We evaluated selected triterpenoid compounds at equal concentrations of 100 μM to compare their potential lipid-reducing activity with the positive control drug (Supplementary Figure S1). Following this initial screening, cycloastragenol was exposed as the most promising in regard to diminished lipid content. Therefore, we extended the concentration range of cycloastragenol supplementation to be between 25 and 200 μM in the glucose-induced obesity model in *C. elegans*. The data from the Nile red lipid staining confirmed a dose-dependent decrease in lipid accumulation as a result of cycloastragenol supplementation until a concertation of 100 μM (Figure 1A,B). Based on the lack of significant difference in the effect on lipid accumulation between the worms supplemented with 100 μM or 200 μM cycloastragenol and the observed reduction in worms’ viability at concentration of 250 μM cycloastragenol (Supplementary Figure S1), we have selected the lower one as the optimal effective concentration to perform all subsequent phenotypic and mechanistic analyses.

Correspondingly, the chemotaxis assay revealed an increase in the preference of cycloastragenol over the vehicle control, which correlates with the increase in the treatment concentration (Figure 1C). This chemoattracting effect of the triterpenoid compound is further an indicator of its safety [29,42]. Next, we evaluated the body parameters for the length, width, and area of the worms in the presence of either orlistat 12 μM or cycloastragenol 100 μM. The automated analysis at the WormLab system detected that cycloastragenol decreased both the width and the body area of the worms to a comparable extent with regard to the positive control orlistat that is corresponding to the observed lipid-reducing effect (Figure 1D–F).

Taken together, the changes in body morphology, the inhibition of glucose-induced lipid accumulation in *C. elegans* obesity model, and the chemoattractant properties of cycloastragenol rationalize further mechanistic analysis in regard to fatty acid metabolism and nutrient-signaling pathways.

### 2.2. Cycloastragenol Downregulates NHR-49/MDT-15 Signaling in C. elegans

The NHR-49/MDT-15 axis controls critical enzymes from the lipid turnover, including the acetyl-CoA-carboxylase (encoded by the *pod-2* gene), the elongases (*elo*), fatty acid desaturases (*fat*), and lipolysis genes such as the patatin-like phospholipase domain-containing protein 1 encoding gene (*atgl-1*) [43,44].

**Figure 2:**
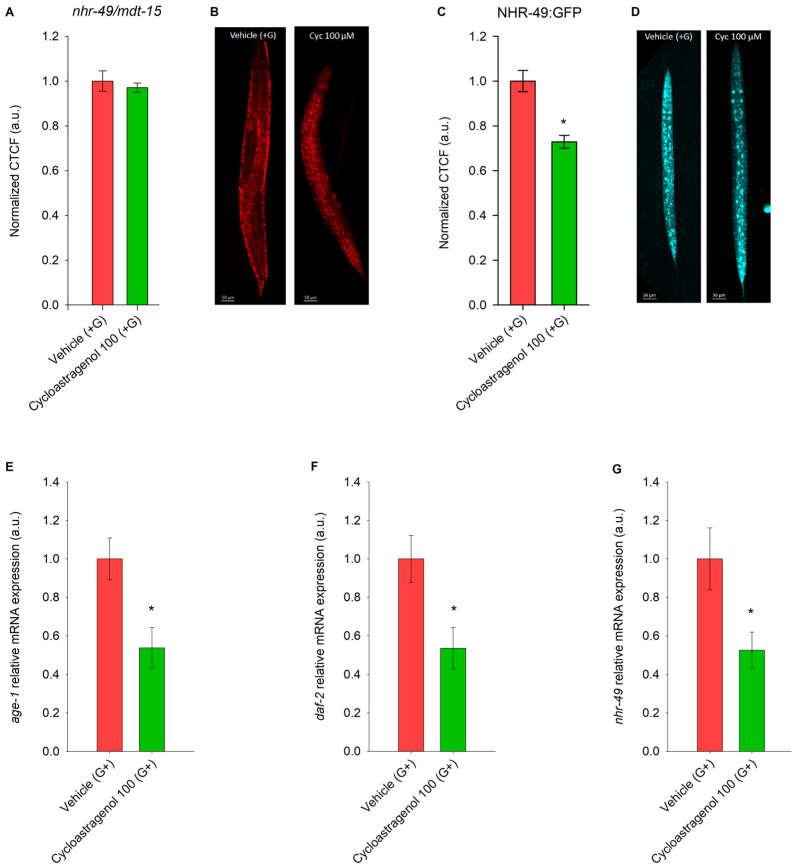
Lipid reduction in glucose-fed nematodes upon cycloastragenol treatment is mediated via NHR-49. (**A**) Quantification of triglyceride accumulation in *nhr-49*/*mdt-15*-deficient strain (QC155 [*nhr-49*(*et8*) I; *mdt-15*(*et14*) III]) upon cycloastragenol (100 μM) application along with glucose (+G) supplementation. Data are represented as corrected total cell fluorescence (CTCF) normalized to the vehicle (+G) group in arbitrary units (a.u.); mean ± SEM, n = 90. (**B**) Representative images of Nile red-stained nematodes, 20× magnification, scale bar (50 μm). (**C**) Quantification of NHR-49 expression upon 100 μM cycloastragenol NHR-49::GFP reporter strain. (**D**) Representative images of the GFP-tagged strain, 20× magnification, scale bar (50 μm). Data from GFP expression are represented as normalized to vehicle (+G) CTCF in arbitrary units (a.u.); mean ± SEM, n = 60. Comparison between groups in (**A**,**C**) was performed using Mann–Whitney rank sum test (* *p* < 0.05). Relative mRNA expression (a.u.) of involved in the IIS pathway (**E**) *age-1*, (**F**) *daf-2*, and (**G**) *nhr-49* represented as mean ± SEM (n = 9) * *p* < 0.05 (*t*-test).

To explore the hypothesis that the anti-obesity potential of cycloastragenol could be mediated through modulation in the IIS, and more specifically through NHR-49 transcriptional activity, we have performed Nile red lipid staining in the double mutant strain deficient in *nhr-49* and *mdt-15* (Figure 2A). The prominent lipid-reducing activity observed in the wild-type N2 strain (Figure 1A) was not replicated in the transgenic strain, suggesting the involvement of both *nhr-49* and *mdt-15* in the cycloastragenol mode of action.

Next, we have explored the NHR-49 transcriptional activity in a GFP-tagged strain and at mRNA level through RT-qPCR analysis. The PHX3258 mutant strain has a GFP tag inserted at the C-terminus of the endogenous nhr-49 locus in *C. elegans*, providing insights into the subcellular dynamics of NHR-49, a key regulator of lipid metabolism. The decrease in the total cell fluorescence (Figure 2C) in the NHR-49::GFP mutant and the downregulation of the respective *nhr-49* gene (Figure 2G) induced by cycloastragenol supplementation indicated that the compound acts as an NHR-49 inhibitor. Correspondingly, the triterpenoid treatment resulted in suppression of key genes from the IIS, including *age-1* (Figure 2E) and *daf-2* (Figure 2F).

Together, these findings highlight that the cycloastragenol anti-obesity effect could be mediated through *daf-2*/*age*-1 suppression and NHR-49 inhibition.

### 2.3. Cycloastragenol Activates AAK-2/SIR-2.1-Mediated Energy Regulation and Intestinal Mitochondria Dynamics in Obesity Model in C. elegans

**Figure 3:**
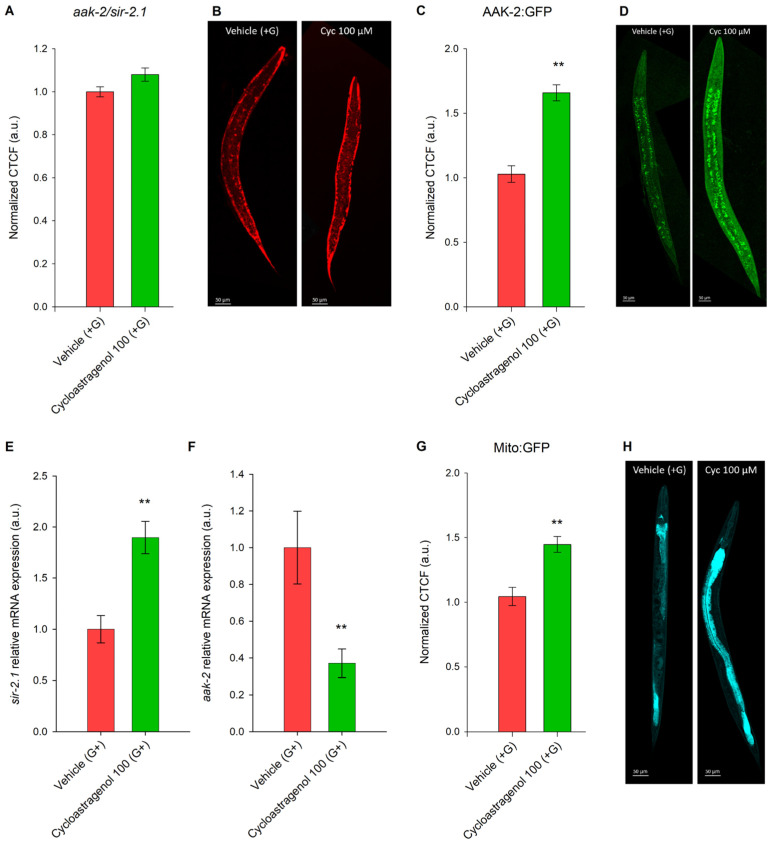
Active AAK-2/SIR-2.1 signaling is necessary for the lipid-reducing effect of cycloastragenol treatment in *C. elegans*. (**A**) Quantification of triglyceride accumulation in *aak-2*/*sir-2.1*-deficient strain MIR13 [*sir-2.1*(*ok434*) IV; *aak-2*(*ok524*) X], upon cycloastragenol (100 μM) application along with glucose (+G) supplementation. Data are represented as corrected total cell fluorescence (CTCF) normalized to the vehicle (+G) group in arbitrary units (a.u.); mean ± SEM, n = 90, Mann–Whitney rank sum test. (**B**) Representative images of Nile red-stained nematodes, 20× magnification, scale bar (50 μm). (**C**) Quantification of AAK-2 expression upon 100 μM cycloastragenol in a GFP reporter strain. Data are represented as normalized to vehicle (+G) CTCF in a.u.; mean ± SEM, n = 60. (**D**) Representative images of GFP-labeled strain nematodes, 20× magnification, scale bar (50 μm). Gene expression (a.u.) of (**E**) *sir-2.1* and (**F**) *aak-2* represented as mean ± SEM (n = 9); ** *p* < 0.01 (*t*-test). (**G**) Quantification of active mitochondria in intestinal tissue expression upon 100 μM cycloastragenol in mito::GFP (SJ4143) reporter strain. (**H**) Representative images of Nile red-stained nematodes, 20× magnification, scale bar (50 μm). Comparison between groups in (**C**,**G**) was performed using *t*-test (** *p* < 0.01).

The AAK-2/SIR-2.1 signaling pathway is intertwined to the IIS and is activated by restricted food availability or as a consequence of increased energy expenditure in insulin-sensitive tissues resulting from exercise, cold, or pharmacological activation [9,18,26]. To validate the hypothesis that the cycloastragenol lipid-reducing effect is dependent on the AAK-2/SIR-2.1 axis, we have performed a Nile red staining in a double mutant strain MIR13 (Figure 3A). Corresponding to the results from the *nhr-49*/*mdt-15*-deficient strain (Figure 2A), the cycloastragenol suppression effect of lipid content observed in the N2 wild-type worms (Figure 1A) was abolished in the *aak-2*/*sir-2.1* mutants (Figure 3A).

Substantial elevation in the AAK-2 protein activity upon cycloastragenol treatment was detected through confocal imaging in a GFP-reporter *C. elegans* strain (Figure 3C). Respectively, the gene expression level of *sir-2.1* was found to be upregulated from cycloastragenol (Figure 3E). Interestingly, the *aak-2* gene was downregulated in the presence of cycloastragenol, which could be due to compensatory feedback regulation resulting from the excessive activation of AAK-2 at a protein level (Figure 3F).

To check the effect of cycloastragenol on mitochondria dynamics, we used the SJ4143 (mito:GFP) strain as a stable transgenic line *C. elegans* expressing GFP in the mitochondria of intestinal cells. The fluorescent detection of mito:GFP intensity disclosed the stimulatory effect on mitochondrial biogenesis in intestinal tissues upon cycloastragenol treatment (Figure 3G,H).

Overall, AAK-2 and *sir-2.1* were found to contribute to the cycloastragenol-induced lipid-reducing activity in the *C. elegans*. Pharmacological agents that increase AAK-2 expression often mimic a state of “perceived starvation” or mitochondrial stress. Notably, the increased AAK-2 expression is positively correlated to the elevated levels of active mitochondria in the intestinal tissue in the worms treated with the triterpenoid compound, suggesting that cycloastragenol supplementation engaged the machinery that *C. elegans* uses to compensate for low energy levels.

### 2.4. Cycloastragenol Acts as a far-3 Enhancer and Potent Inhibitor of FAT-7 to Modulate Fatty Acid Metabolism

**Figure 4:**
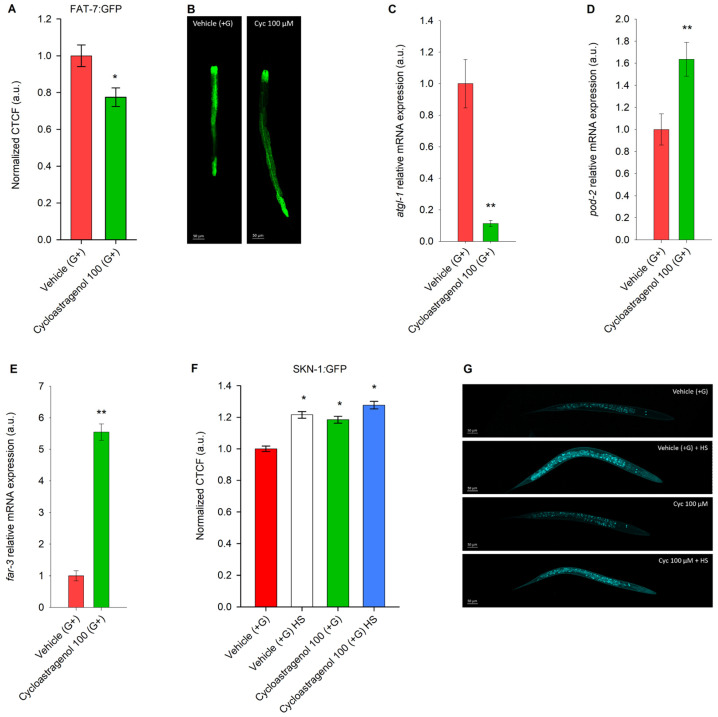
Gene expression analysis exposed cycloastragenol as a *far-3* enhancer and potent inhibitor of FAT-7 in the obesity model in *C. elegans*. (**A**) Expression of FAT-7 upon cycloastragenol (100 μM) application along with glucose (+G) supplementation in GFP-labeled strain (DMS303). Data are represented as CTCF in arbitrary units (a.u.), mean ± SEM, n = 60, * *p* < 0.05 (Mann–Whitney rank sum test). (**B**) Representative images of FAT-7::GFP strain, 20× magnification, scale bar (50 μm). Gene expression (a.u.) of (**C**) *atgl-1*, (**D**) *pod-2*, and (**E**) *far-3* represented as mean ± SEM (n = 9); ** *p* < 0.01 (*t*-test). (**F**) Quantification of SKN-1 expression upon 100 μM cycloastragenol application along with glucose (+G) supplementation with or without heat stress (HS) stimulation in SKN-1::GFP reporter strain (LD1). Data for GFP expression are represented as normalized to vehicle (+G) CTCF in arbitrary units (a.u.); mean ± SEM, n = 60, and * *p* < 0.05 (ANOVA on ranks). (**G**) Representative images of SKN-1::GFP nematodes, 20× magnification, scale bar (50 μm).

Transcription factors from the nuclear hormone receptor family, especially NHR-49 and NHR-80, are important for the modulation the ∆9 desaturases *fat-2*, *fat-5*, *fat-6*, and *fat-7* [15,19]. The *fat-7* gene encodes an enzyme that is critical for the precise regulation of ∆9 desaturation, securing proper membrane fluidity and nutrient utilization, and could be influenced by various environmental conditions, such as temperature shifts and nutritional availability [19,45]. Treatment with cycloastragenol suppressed FAT-7 translational activity (Figure 4A,B) and the mRNA expression level of the *atgl-1* gene that encodes a key lipolysis enzyme in *C. elegans* (Figure 4C). The gene *pod-2* that encodes the acetyl-CoA-carboxylase homolog in *C. elegans*, a rate-limiting enzyme in the acetyl-CoA to manonyl-CoA transition, was found to be upregulated by the cycloastragenol supplementation (Figure 4D). These observations correspond to the downregulation of the *daf-2/age-1* genes and the suppression in the NHR-49 transcriptional activity that were observed in the results of the triterpenoid treatment (Figure 2).

The *far-3* gene in *C. elegans* encodes the orthologue of the fatty acid and retinol-binding protein that regulates lipid metabolism, transport, and stress responses, especially in response to high glucose levels and nutritional overload. Studies exposed that it is involved in protecting against glucose-induced lifespan shortening [43,46,47]. Our data indicated the upregulation in *far-3* gene expression levels by over 5-fold in response to cycloastragenol supplementation in the glucose-induced model in *C. elegans* (Figure 4E).

The SKN-1 protein, the orthologue of the mammalian NRF-2, is a master regulator of the oxidative stress response and detoxification functions in *C. elegans*. Moreover, SKN-1 coordinates in mitochondrial biogenesis and takes a central role in the starvation response [22,25]. We have employed the LD1 reporter mutant strain that reflects the SKN-1 transcriptional activation in response to cellular stress. Supplementation with cycloastragenol has stimulated SKN-1 activity to an extent that is comparable to the positive control group that was exposed to heat stress (Figure 4F,G).

Taken together, the observed reduction in FAT-7 desaturase activity reflects potent NHR-49 inhibition and corresponds to diminished lipid accumulation in wild-type worms. Cycloastragenol induced SKN-1 activation, hinting towards the modulation of the oxidative stress response. Finally, the overexpression of the *far-3* gene could be a key part of the overall cycloastragenol-induced healthspan improvement and glucotoxicity prevention in *C. elegans*.

## 3. Discussion

Obesity represents a global health challenge that affects a substantial proportion of the world’s population. According to recent epidemiological data, approximately 650 million adults are affected by obesity. This chronic metabolic condition is characterized by excessive accumulation of body fat, leading to an increased risk of numerous diseases, including type 2 diabetes, cardiovascular disorders, chronic kidney disease, site-specific cancers, musculoskeletal impairments, infections, fertility problems, and mental health disorders [1,2,7]. In addition to its pathological and morphological manifestations, being overweight negatively influences the quality of life, social functioning, and the psycho-emotional state of the individual. Given the increasing prevalence of obesity and its multifaceted consequences, developing effective therapeutic approaches and prevention strategies are key priorities [6]. In this regard, natural compounds play an important role in targeting obesity mechanisms by modulating various signaling pathways; hence, there is considerable scientific interest in their investigation [20,28,48].

Cycloastragenol has been implicated in the preservation of telomere function [32,34,38,49], the attenuation of senescence-associated phenotypes [25,50], anti-fibrotic effects [30,36], and the promotion of autophagy [35,51] or, depending on the cellular context, apoptosis [37,52]. Cycloastragenol safety has been evaluated in numerous pharmacological models [32,33,40] that have found no significant rise in neoplasia incidence [49], no treatment-related deaths, and no evidence of genotoxicity or subchronic toxicity even at the high doses of 150 mg/kg/d in mice for over 90 days [53]. Human data from long-term use of a cycloastragenol-containing supplement also reported very few adverse effects, none of which were attributed to the cycloastragenol itself [54]. These data certify the relevance to human health of these investigations that validate the potential of cycloastragenol to modulate lipid metabolism and obesity.

**Figure 5:**
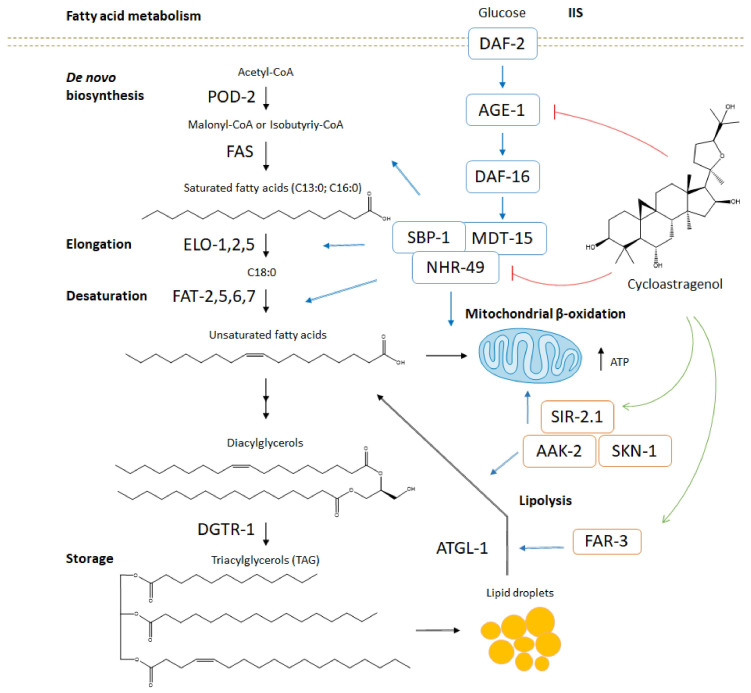
Proposed molecular model of the anti-obesity effect of cycloastragenol in *C. elegans*. Nutritional overload elevates insulin, which, upon binding to the insulin receptor, amplifies insulin receptor substrate levels. Insulin triggers de novo lipid biosynthesis through insulin/insulin growth factor receptor signaling (IIS), leading to the activation of the phosphatidylinositol 3-kinase (AGE-1) and the upregulation in specific lipogenic transcription factors. Subsequently, the activated protein kinase B transmits the signal to the mammalian target of rapamycin, which restrains the Forkhead box protein O (DAF-16) nuclear translocation and stimulates lipogenesis by the upregulation of key molecular regulators such as the nuclear hormone receptor family member 49 (NHR-49), the mediator of RNA polymerase II transcription subunit 15 (MDT-15), and the sterol regulatory element binding protein (SBP). Alternatively, calorie restriction, metformin, and some (phyto)chemicals that change the adenosine monophosphate (AMP)/adenosine diphosphate (ADP) ratio or the nicotinamide adenine dinucleotide (NAD+) levels activate the 5′-AMP-activated protein kinase catalytic subunit alpha-2 (AAK-2) and the NAD-dependent protein deacetylase (SIR-2.1) pathway as major cellular energy sensors to induce autophagy, lipolysis, mitochondrial biogenesis, and fatty acid oxidation. Cycloastragenol reduces glucose-induced lipid accumulation in *C. elegans* through potent inhibition of the NHR-49 signaling pathway, resulting in FAT-7 reduction, *far-3* upregulation, and diminished lipid content. Simultaneously, cycloastragenol supplementation activates the AAK-2/SKN-1 axis and elevates intestinal mitochondrial activity. Black arrows are representative for the critical steps of the fatty acid metabolism in *C. elegans*; the blue arrows are representative for the molecular signaling networks of the IIS and AAK-2/SIR-2.1 pathways; the green arrows represent the proposed molecular targets that are activated by cycloastragenol and red lines represent the proposed targets that are inhibited by cycloastragenol.

Our findings provide insights into the molecular mechanism of its lipid-reducing activity in glucose-induced obesity model in *C. elegans*. This model is invaluable for understanding how conserved signaling pathways influence metabolism, offering insights that could lead to novel therapeutic strategies against obesity. Furthermore, its unique characteristics, including the presence of the necessary genes for fatty acid metabolism and mitochondrial function, position it as an unparalleled model for lipid metabolism studies, advancing our understanding of how genetic factors influence healthspan and the development of obesity [13,14,18,24,25,26,27]. In our study, the supplementation of glucose-fed worms with the triterpenoid resulted in the downregulation of NHR-49 transcriptional activity, and the enhancement of the AAK-2 and SKN-1 activity. A schematic representation of the proposed molecular model of action of cycloastragenol is provided in Figure 5.

The NHR-49 and MDT-15 proteins form a transcriptional complex that regulates lipid metabolism, coordinates stress responses, and orchestrates gene expression of the fatty acid de novo biosynthesis, elongation, desaturation, and lipolysis [9,17,20]. Our phenotype analysis revealed changes in body morphology, inhibition of glucose-induced lipid accumulation, and a chemoattractant effect upon cycloastragenol supplementation in the *C. elegans* obesity model, which exceeded the effect of the positive control drug, orlistat. These findings justified the evaluation of the potential involvement in the cycloastragenol mode of action of IIS signaling-related genes that control the fatty acid metabolism and nutrient-signaling pathways. Earlier studies on the cycloastragenol (25 μM) reported the selective activation of the FXR in HepG2 cell line, but not of peroxisome proliferator-activated receptors (PPARs). In their in vivo experiment, Gu et al. [39] found lipid-reducing activity in a diet-induced obesity NAFLD model in mice that was mediated by FXR selective activation in hepatic tissue. Interestingly, the gene expression analysis of the liver exposed downregulation in PPAR beta, but upregulation in PPAR alpha and gamma subtypes in the NAFLD model [39]. Our data exposed cycloastragenol as a potent inhibitor of the transcriptional activity of NHR-49 (the orthologue of the PPARs in *C. elegans*), which is correlated with the downregulation of the *daf-2* and *age-1* genes from the IIS pathway.

Given that the IIS is critical for the regulation of fatty acid metabolism, we focused on examining fatty acid desaturation factors. The FAT-7 desaturase induces the biotransformation of stearic acid to oleic acid, and elevated oleic acid levels are found to suppress *fat-7* expression. This process is highly sensitive to modification of diet composition and supplementation with natural compounds such as resveratrol [55]. Recent studies reported the results from a genome-wide *Escherichia coli* mutant screen to investigate the microbial factors that modulate *C. elegans* FAT-7 levels. Das et al. [19] identified several *E. coli* mutant strains that reduced FAT-7 expression and investigated their effects on the host lifespan. Notably, all the *E. coli* mutants that inhibited FAT-7 exhibited lifespan-extending effects in *C. elegans* [19]. The detected reduction in FAT-7 activity in our experiments reflects the potential of cycloastragenol to improve healthspan and its ability to diminish glucose-induced lipid accumulation.

Mechanistic studies that explore the glucose-induced lifespan shortening in *C. elegans* utilize the *far-3* promoter (far-3p::GFP) as an in vivo glucose reporter [43,56]. The findings from a recent study confirmed that glucose toxicity results from the accumulation of saturated fatty acids and suggested that *far-3* may mediate metabolic remodeling by increasing the disaccharide trehalose and decreasing the polysaccharide glycogen, ultimately leading to prolonged lifespan and healthspan [56]. In our study, considerable overexpression of the *far-3* gene was found, which was positively correlated with downregulation in the *atgl-1* resulting from cycloastragenol supplementation. The *far-3* enchaining activity might be a critical part of the overall healthspan improvement and the diminished glucotoxicity in *C. elegans* that is induced by cycloastragenol. Suppressed *atgl-1* expression resulting from a high-glucose diet has been shown to augment mitochondrial oxidation and stress, as the organism shifts toward lipid storage rather than mobilization. During starvation, nutrient depletion stimulates lipolysis through AMPK/SIRT signaling activation and liberates the suppression of *atgl-1* expression [19,56]. Despite the fact that the cycloastragenol-mediated inhibition of *atgl-1* may appear controversial, it could represent a biological effect on the metabolic profile that shifts it towards suppressing lipolytic mRNA levels as part of a broader metabolic reorganization. Similar observations have been reported in our earlier study on the anti-obesogenic effect of betulinic acid in *C. elegans* [20]. Furthermore, the cycloastragenol-induced upregulation of *pod-2* that we observed may be associated with metabolic adaptations to changes in nutritional availability, as well as with the lifespan-extending effect of IIS inhibition and the subsequent activation of DAF-16 [29,57].

Mitochondria are efficient, highly adaptable bioenergetics organelles that power the energy metabolism of the cell and organism. Mitochondrial structure and function are dynamically tuned in response to tissue-specific energy demands, growth, and development [8,22,24]. Together, AAK-2/SIR-2.1 interact to fine-tune the energy balance, autophagy, and lipid utilization in mitochondria [4,13,17,18]. Furthermore, the favorable metabolic effects of the AAK-2 activation are commonly associated with the overexpression of sirtuins known to promote extended lifespan in *C. elegans* [21]. Finally, the positive feed-forward loop formed between AAK-2/SIR-2.1 stimulates the oxidative stress response in the mitochondria through upregulation in SKN-1 activity [4,22,25]. Our study uncovers the central role of AAK-2 activation and *sir-2.1* upregulation in the cycloastragenol-induced anti-obesity effect in glucose-fed *C. elegans*. This is consistent with recent studies reporting autophagy promotion upon cycloastragenol treatment through the AMPK/ULK1/mTOR pathway in human non-small-cell lung cancer lines [37] or via Notch1 signaling in human keratinocytes [51].

In *C. elegans*, the SKN-1 activation is known to modulate the oxidative stress response, lysosomal lipolysis, mitochondrial biogenesis, and mitophagy [9,22]. Cycloastragenol improved renal function through enhanced mitophagy in *db/db* mice and HK-2 cells by stimulating the TFEB-mediated autophagy. The triterpenoid treatment has been reported to reduce the accumulation of mitochondrial ROS, to enhance mitochondrial membrane potential, and to promote mitophagy and mitochondrial biogenesis [40]. Notably, cycloastragenol-induced AAK-2 activation is positively correlated with increase in the SKN-1 transcriptional activity and the rise in active intestinal mitochondria.

## 4. Materials and Methods

### 4.1. Materials

Nematode growth medium (NGM; #MBS652667) was obtained from MyBiosource Inc. (San Diego, CA, USA). Cycloastargenol (#HY-N1485; purity ≥ 98%; molecular weight 490.72 g/M), roburic acid (#HY-N0481; purity ≥ 99%; molecular weight 440.70 g/M), gypenoside XLIX (#HY-N1990; purity ≥ 99%; molecular weight 1047.23 g/M), momordin Ic (HY-N0330; purity ≥ 99%; molecular weight 764.94 g/M), and pulchinenoside C (#HY-N0205; purity ≥ 99%; molecular weight 1221.38 g/M) were purchased from MedChemExpress (Medchemtronica AB, Stockholm, Sweden). Orlistat (#O4139; purity ≥ 98%; molecular weight 495.7 g/M), LB broth Lennox (#L3022), agar powder (#05039), M9 minimal salts (#M6030), fluoroshield histology mounting medium (#F6182), Nile red (#72485), sodium hydroxide, and glucose were supplied from Sigma-Aldrich Co. (St. Louis, MO, USA). The reagents for RNA isolation, quantitative real-time polymerase chain reaction (RT-qPCR) analysis, as well as the consumables used for gel electrophoresis, were supplied from Bio-Rad Laboratories Inc. (Hercules, CA, USA).

### 4.2. Caenorhabditis Elegans: Maintenance and Treatment

Strains used in this study were obtained by the *Caenorhabditis* Genetic Centre (CGC, University of Minnesota, Minneapolis, MN, USA), which is funded by NIH Office of Research Infrastructure Programs (P40 OD010440). The nematodes were maintained according to standard procedures on NGM plates at 20 °C using *E. coli* strain OP50 as a food source. All the experiments were performed following the recommendations for transparent reporting in animal research, including model organisms such as *C. elegans*, in accordance with the guidelines for Animal Research: Reporting of In Vivo Experiments (ARRIVE vol. 2.0). The *C. elegans* strains used were as follows: N2 wild-type Bristol strain, MIR13 [*sir-2.1*(*ok434*) IV; *aak-2*(*ok524*) X], QC155 [*nhr-49*(*et8*) I; *mdt-15*(*et14*) III]; AGD467 *uthEx490* [*aak-2*(*intron 1*)::*aak-2*(*aa1-aa321 T172D*)::GFP + *crtc-1p*::*crtc-1*::tdTomato + *rol-6*(*su1006*)]; DMS303 *nls590* [*fat-7p*::*fat-7*::GFP + *lin15*(+)] V; PHX3258 [*nhr-49*(*syb3258*[*nhr-49*::GFP]) I]; SJ4143 *zcIs17* [*ges-1*::GFP(*mit*)]; and LD1 *ldIs7* [*skn-1b/c*::GFP + *rol-6*(*su1006*)]. The experiments employed worm populations synchronized to the same developmental stage, achieved through a standard bleaching protocol. All of the experimental treatments were added to heat-inactivated and 10-fold concentrated *E. coli* OP50. To model the increased lipid accumulation the NGM plates, they were supplemented with 2% glucose [20], and normal *E. coli* OP50 was used as a food source until the L2 stage was reached. Then, the worms were transferred to fresh NGM plates with glucose, respectively, and the heat-inactivated *E. coli* was supplemented with vehicle (to a final concentration of 0.2% DMSO), orlistat 12 μM, roburic acid 100 μM, gypenoside XLIX 100 μM, momordin Ic 100 μM, pulchinenoside C 100 μM, or cycloastragenol (25, 50, 100, and 200 μM) for the following 24 h.

### 4.3. Lipid Accumulation Assay

The staining method for detecting fat depositions was conducted following previously established protocols employing Nile red dye [20,58]. Approximately 1000–1500 L4 larvae from each experimental group were collected and washed with M9 buffer prior to fixation with 40% isopropanol for 3 min. Subsequently, the fixative was removed, and the staining solution was applied for a 2 h incubation period. Imaging of the lipid depositions was performed at 10× or 20× magnification using the confocal system Stellaris 5 with an inverted microscope DMi8 from Leica (Wetzlar, Germany). For each experimental group, quantification of the fluorescence intensity of randomly selected nematodes (n ≥ 90) was conducted using ImageJ software version 1.53t, normalized to the vehicle group. The results were presented as normalized corrected total cell fluorescence (CTCF) in arbitrary units (a.u.).

### 4.4. Chemotaxis Assay

The chemotaxis assay was conducted in accordance with previously established protocols [21]. The Petri dish was partitioned into four quadrants, marked as the test (“T”) or control (“C”) zone. Two-microliter drops of both the control (*E. coli* OP50) and respective experimental treatments were placed in the respective quadrants at equidistant spots from the center. A circular area with a diameter of 1 cm was drawn at the center of the dish, onto which 100–120 L4 nematodes in the M9 buffer were placed. Following a 1 h incubation period at 20 °C, the Petri dish was transferred to 4–6 °C for 30 min to immobilize the nematodes, facilitating subsequent scoring. The worms within each quadrant of the Petri dish were counted, and the chemotaxis index (CI) was determined using the following equation: CI = (Quadrant test area 1 + Quadrant test area 2) − (Quadrant control area 1 + Quadrant control area 2), divided by the total number of nematodes.

### 4.5. Phenotype Analysis

The individual body area, length, and width were recorded using an automated nematode tracking system, WormLab system (MBF Bioscience, Williston, VT, USA), as previously described [58]. Late L4-stage larvae, pre-treated with the 100 μM cycloastragenol, 12 μM orlistat, or vehicle for 24 h, were then observed. For each group, a video was recorded for 1 min and subsequently analyzed with the WormLab software (version 2023.1.1, MBF Bioscience, Williston, VT, USA).

### 4.6. Gene Expression Analysis Through RT-qPCR

Total RNA was extracted from approximately 3000–4000 L4 larvae per experimental group using PureZol (#7326890; Bio-Rad) after a 24 h incubation period with the experimental treatments. The integrity and quantity of the extracted RNA were assessed using agarose gel electrophoresis and UV spectroscopy. Reverse transcription was carried out using the First Strand cDNA synthesis kit (#MB12502 NZYtech, Lisbon, Portugal). The expression levels of mRNAs were quantified using the ΔΔCT method with CFX Maestro software version 4.1.2433.1219 using SsoFast EvaGreen Supermix for qPCR reaction (Bio-Rad). The *pmp-3* and *iscu-1* were used as reference genes, and the results were normalized to the vehicle group. The data were presented as normalized relative mRNA expression in arbitrary units from three independent biological experiments. The nucleotide sequences of the primers, used for analysis of relative mRNA expression, are provided in Supplementary Table S1.

### 4.7. Confocal Imaging of Reporter Transgenic Strains

The transgenic age-synchronized nematodes (around 1000–1500) of the GFP-tagged strains were treated and mounted on glass slides containing levamisole (5 mM) as anesthesia. Images of the worms were acquired at 10× or 20× magnification using the GFP filter in the Leica Stellaris 5 confocal system with an inverted microscope DMi8. For each experimental group, the quantification of fluorescence intensity of randomly selected nematodes (n ≥ 60) was performed using ImageJ software version 1.53t, normalized to the vehicle group. The results were presented as normalized CTCF in arbitrary units (a.u.).

### 4.8. Statistical Analysis

The obtained results were subjected to analysis in SigmaPlot v11.0 from Systat Software GmbH (Erkrath, Germany). Each assay was performed in three independent biological experiments and the data were represented as mean ± standard error of the mean (SEM). The Shapiro–Wilk was used as a normality test to assess the data distribution. The statistical significance between two groups was determined by a *t*-test and for multiple comparisons the one-way analysis of variance (ANOVA) test, followed by Tukey’s post hoc test, denoted as * *p* < 0.05, ** *p* < 0.01, compared to the glucose-supplemented (+G) vehicle group, respectively. When the normality test failed, the comparison between two groups was performed by the Mann–Whitney rank sum test. For multiple comparisons, ANOVA on ranks, followed by Dunn’s post hoc test, was applied. Confocal microphotographs of the lipid staining and GFP-reporter strains are representatively selected amongst the images from three independent experiments.

## 5. Conclusions

The development of effective therapeutic and preventive strategies hinges upon a deep understanding of the underlying molecular pathways regulating energy metabolism, such as the PI3K/AKT, the AMPK/SIRT, and the NRF2 signaling cascades. Our findings highlight that cycloastragenol exhibits promising anti-obesity effects in *C. elegans* by modulating these critical pathways. These observations indicate that the pentacyclic triterpenoid compound cycloastragenol exerts its anti-obesity action through potent inhibition of NHR-49 transcriptional activity, resulting in reduced FAT-7 expression and diminished lipid content. Simultaneously, cycloastragenol supplementation stimulates the AAK-2 and SKN-1 signaling pathways, enhances *far-3* expression, and elevates intestinal mitochondrial activity. These insights facilitate anti-obesity drug development and advance dietary interventions (e.g., dietary supplements or functional foods) for weight control.

## Data Availability

The original contributions presented in this study are included in the article and Supplementary Materials. Further inquiries can be directed to the corresponding author.

## Supplementary Materials

The following supporting information can be downloaded at: https://www.mdpi.com/article/10.3390/ijms27020772/s1.
