# Supplementary material for: Cycloastragenol Improves Fatty Acid Metabolism Through NHR-49/FAT-7 Suppression and Potent AAK-2 Activation in Caenorhabditis elegans Obesity Model
Source: Int J Mol Sci. 2026 Jan 13;27(2):772. doi: 10.3390/ijms27020772 (PMC12841531; doi:10.3390/ijms27020772)
Supplement: Supplementary file 1 [file ijms-27-00772-s001.zip › ijms-4082573-supplementary.pdf]

Supplementary material

Cycloastragenol improves fatty acid metabolism through NHR-49/FAT-7 suppression and potent AAK-2 activation in *Caenorhabditis elegans* obesity model

Liliya V. Mihaylova[1,2,*], Martina S. Savova[1,2], Monika N. Todorova[1], Valeria Tonova[3], Biser K. Binev[1,2], Milen I. Georgiev[1,2]

[1] Laboratory of Metabolomics, Department of Biotechnology, Institute of Microbiology, Bulgarian Academy of Sciences, 139 Ruski Blvd., 4000 Plovdiv, Bulgaria

[2] Department of Plant Cell Biotechnology, Center of Plant Systems Biology and Biotechnology, 4000 Plovdiv, Bulgaria

[3] Department of Molecular Stress Physiology, Center of Plant Systems Biology and Biotechnology, 4000 Plovdiv, Bulgaria

[*] Correspondence: Assoc. Prof. Dr. Liliya V. Mihaylova, Laboratory of Metabolomics, Department of Biotechnology, Institute of Microbiology, Bulgarian Academy of Sciences, 139 Ruski Blvd., 4000 Plovdiv, Bulgaria and Department of Plant Cell Biotechnology, Center of Plant Systems Biology and Biotechnology, 4000 Plovdiv, Bulgaria; e-mail: liliya.vl.mihaylova@gmail.com; Tel.: +359-32-64-24-30.

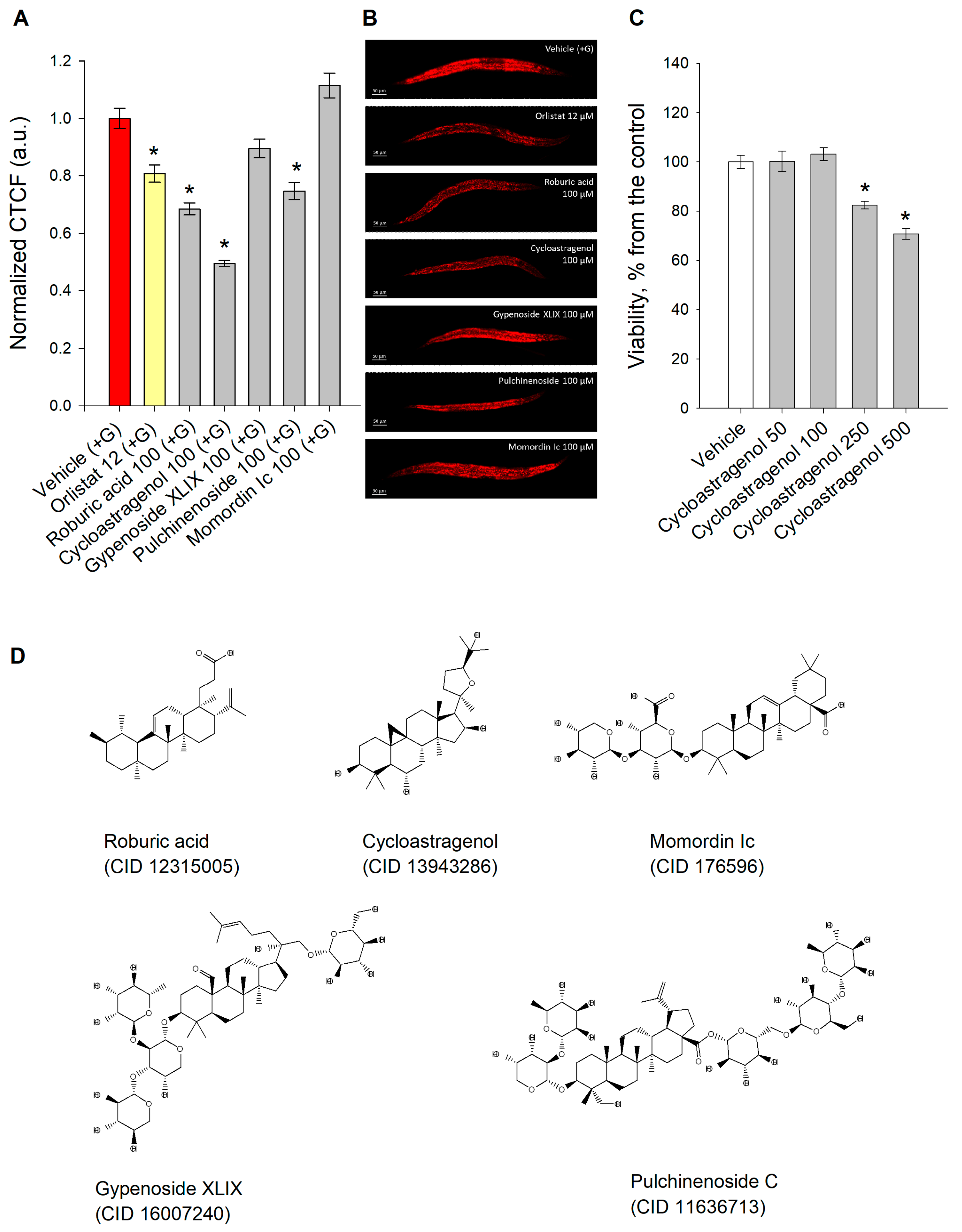

Supplementary Figure S1. Lipid accumulation (A), representative confocal images at magnification 20x; scale bar 50 µm; (B), Viability assay provided rationale for the safety of the selected concentrations of cycloastragenol; n = 12 (C) and chemical formulas with PubChem CID numbers of the evaluated triterpenoid natural compounds (D) in the glucose-induced

obesity model in *Caenorhabditis elegans*. Data are presented in mean ± SEM, $*p \leq 0.05$. For comparison between the groups one-way ANOVA on Ranks, followed by Dunn's *post hoc* test were used.

Supplementary Table S1. Primer sequences used in the RT-qPCR of mRNAs.

Gene	Forward (5′ to 3′)	Reverse (5′ to 3′)
aak-2	GGACGTCATTGCTCACGAGTGG	ATAGCGCTCAGTGACCTCTCGG
age-1	GACCGAGAATGGCAAAGGATCG	GCACATTTGCAAGAAACCACGG
atgl-1	CTGCGGCTGTATCTGTTCCAAC	TTCTCAAAATCGCTGCTGGCTC
cebp-2	AGCGATGAGTGGAAATCGGAAGCG	GTCGGGTTCTGTTCACAGCTTCGT
daf-2	GAGACACGATGCGAGTGAGACG	GGATCAGCGGCTTCTTTCCACC
far-3	TCAACGTTTTCTGCTTGGTTCT	TGGAAGGAGATCTTTGTGTTGC
fat-2	GGTCTACGAAGCTGATGAGTGGG	GGCGACGTGACCGTTGGTAATG
iscu-1	TCGCTTCAAATCAGTTCAGCCG	GACATTTCGCGGGTTCTCGTAG
nhr-49	TCCGAGTTCATTCTCGACG	GGATGAATTGCCAATGGAGC
pmp-3	AGTGGAGAATGGTCCCTTCACG	ATGGGAGGGGGAACGTGATTAC
pod-2	GGAGGCGATGAATACGATGTGC	AGGCGTTGTTGATGAAGTCAGC
sbp-1	TGTTTTCGGTTGGCTGGGAATG	CAAACGACTGAGACAGGCTTCG
sir-2.1	TGTGTTTGTTTCGGGTGCATCGG	AGAAGTTGCGGTCACACACGGG
skn-1	TTCCGCGTCGACGAATCTTGCG	AGCTTCCAGTGTCGGCGTTCCA
